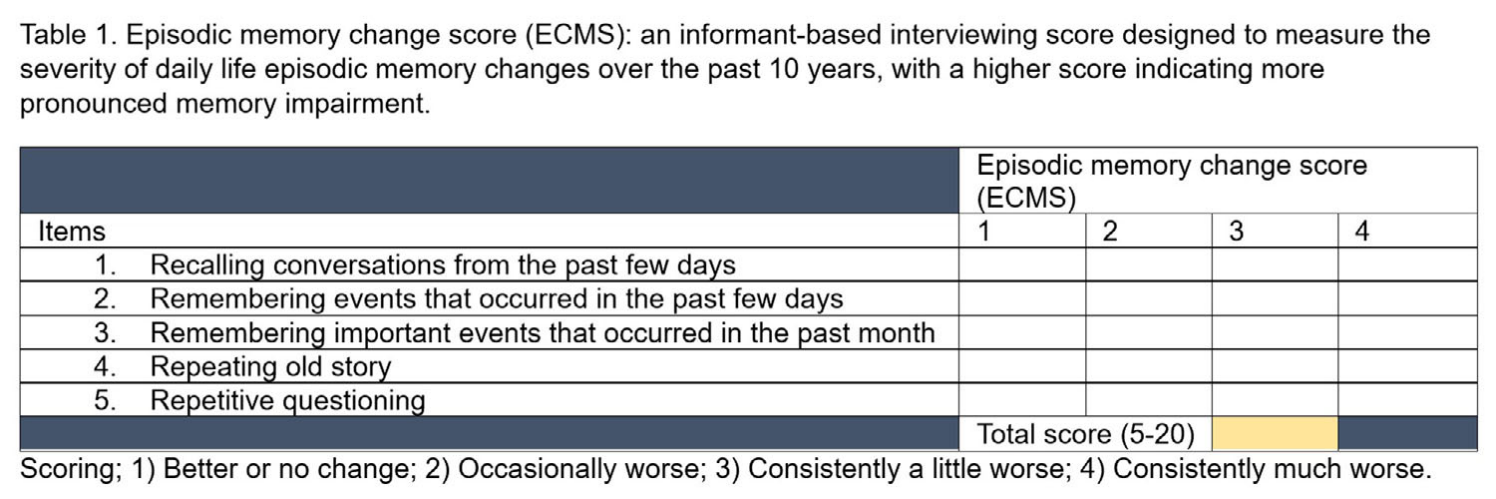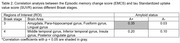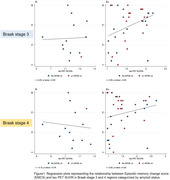# Everyday life episodic memory change and limbic quantitative PI‐2620 tau PET in participants with mild cognitive impairment and mild dementia

**DOI:** 10.1002/alz.085860

**Published:** 2025-01-03

**Authors:** Kittithatch Booncharoen, Akarin Hiransuthikul, Poosanu Thanapornsangsuth, Sekh Thanprasertsuk, Chanan Sukprakun, Supatporn Tepmongkol, Kammant Phanthumchinda, Yuttachai Likitjaroen

**Affiliations:** ^1^ Neurocognitive Unit, Division of Neurology, Faculty of Medicine, Chulalongkorn University, Bangkok Thailand; ^2^ Memory Clinic, King Chulalongkorn Memorial Hospital, Bangkok Thailand; ^3^ Memory Clinic, Division of Neurology, King Chulalongkorn Memorial Hospital, Bangkok Thailand; ^4^ Department of Preventive and Social Medicine, Faculty of Medicine, Chulalongkorn University, Bangkok Thailand; ^5^ Thai Red Cross Emerging Infectious Diseases Health Science Centre, King Chulalongkorn Memorial Hospital, Bangkok Thailand; ^6^ Cognitive, Clinical and Computational Neuroscience (CCCN) Research Unit, Chulalongkorn University, Bangkok Thailand; ^7^ Chula Neuroscience Center, King Chulalongkorn Memorial Hospital, Bangkok, Bangkok Thailand; ^8^ Division of Nuclear Medicine, Department of Radiology, Faculty of Medicine, Chulalongkorn University, Bangkok Thailand

## Abstract

**Background:**

Episodic memory change is among the earliest symptoms of Alzheimer’s disease (AD). Accumulation of neurofibrillary tangles in limbic regions serves as a key indicator of the initial tau abnormality. This study aimed to investigate the correlations between episodic memory change and quantitative tau deposition specifically in Braak stage 3‐4 Limbic regions among participants with mild cognitive impairment (MCI) and mild dementia.

**Method:**

Participants diagnosed with MCI and mild dementia were recruited from the memory clinic at King Chulalongkorn Memorial Hospital, Thailand. Episodic memory change was assessed using the Episodic Memory Change Score (EMCS), an informant‐based interviewing score designed to measure the severity of daily life episodic memory changes over the past 10 years, with a higher score indicating more pronounced memory impairment (Table 1). Participants underwent amyloid (Florbetaben) and tau (PI‐2620) positron emission tomography (PET) scans. amyloid PET results were defined as either amyloid‐negative (A‐) or amyloid‐positive (A+). Quantitative tau deposition was measured using the Standardized Uptake Value Ratio (SUVR). Correlations between EMCS and Braak stage 3‐4 tau SUVR were performed using Pearson’s correlation base on amyloid status (A+ and A‐).

**Result:**

Among the 50 enrolled participants (median [interquartile range] age 72 [65‐77] years), 37 (74%) exhibited Alzheimer’s pathological changes (A+). Table 2 presents the correlation analysis between the EMCS and tau SUVR across different Braak stages. In Braak stage 3, higher EMCS significantly correlated with higher tau SUVR in A+ participants (r = 0.35, p = 0.03), but not in A‐ participants (r = 0.03, p = 0.92). In Braak stage 4, a correlation was noted, but did not reach statistical significance, between higher EMCS and increased tau SUVR in A+ participants (r = 0.20, p = 0.23) (Figure 1).

**Conclusion:**

In participants with Alzheimer’s pathologic change, we found a significant correlation between episodic memory impairment and tau abnormality in Braak stage 3 regions, which encompass critical elements of the Papez circuit. The results suggest that EMCS could serve as a practical and easily accessible tool for early detection of tau PET positivity in A+ individuals, offering a straightforward approach for identifying Alzheimer’s‐related tau changes in a timely manner.